# Generation of Steam by Heated Metal

**Published:** 1831

**Authors:** 


3. *Generation of Steam by Heated Metal.*—In boilers of high pressure engines, the heat applied to a part not containing water, sometimes raises that part of the boiler to a dull read heat, which suddenly coming in contact with a portion of contained water, generates steam with such rapidity, as to burst the boiler. Mr. Johnson, of Philadelphia, made a number of experiments, with a view to procuring a remedy for this defect. He found by immersing iron, raised to different temperatures, in boiling water, that more steam was generated in a given time, by iron of a red heat, just visible in daylight, than by the same piece of iron raised to a white heat. This was thought to arise from the greater quantity of steam forming an atmosphere around the white hot iron, and thus preventing the water from coming in contact with the iron. The steam generated, bears a direct relation to the weight of the metal, being about one pound of steam for every nine pounds of iron. In comparing cast iron with malleable, it was found that cast iron raised to the same temperature, generates more than wrought iron, being about one pound of steam for every eight pounds and a quarter of iron.—*Silliman's Journal, Vol. XIX. p.* 292.

